# Possible Underlying Mechanisms for the Renoprotective Effect of Retinoic Acid-Pretreated Wharton’s Jelly Mesenchymal Stem Cells against Renal Ischemia/Reperfusion Injury

**DOI:** 10.3390/cells11131997

**Published:** 2022-06-22

**Authors:** Mai Barakat, Abdelaziz M. Hussein, Mohamed F. Salama, Amira Awadalla, Nashwa Barakat, Mohamed Serria, Mohamed El-Shafey, Mohamed El-Sherbiny, Mohamed A. El Adl

**Affiliations:** 1 Department of Biochemistry, Faculty of Veterinary Medicine, Mansoura University, Mansoura 35516, Egypt; mai.barakat@aucegypt.edu (M.B.); mfisalama@gmail.com (M.F.S.); mohamedel_adl@mans.edu.eg (M.A.E.A.); 2 Institute of Global Public Health and Human Ecology, School of Science and Engineering, American University, Cairo 11835, Egypt; 3 Department of Medical Physiology, Faculty of Medicine, Mansoura University, Mansoura 35516, Egypt; 4 Urology and Nephrology Center, Mansoura University, Mansoura 35516, Egypt; a.lahlouba@hotmail.com (A.A.); nashwab2006@yahoo.com (N.B.); 5 Department of Biochemistry, Faculty of Medicine, Mansoura University, Mansoura 35516, Egypt; sorriaeg@mans.edu.eg; 6 Department of Anatomy and Embryology, Faculty of Medicine, Mansoura University, Mansoura 35516, Egypt; mmelshafey@fcms.edu.sa; 7 Physiological Sciences Department, Fakeeh College for Medical Sciences, Jeddah 21461, Saudi Arabia; 8 Department of Basic Medical Sciences, College of Medicine, AlMaarefa University, Riyadh 71666, Saudi Arabia; msharbini@mcst.edu.sa

**Keywords:** retinoic acid, WJ-MSCs, Wnt/β-catenin, renal ischemia

## Abstract

Objectives: The current work investigated the effect of Wharton jelly mesenchymal stem cells (WJ-MSCs) pretreated with all-trans-retinoic acid (ATRA) on renal ischemia in rats and the possible role of oxidative stress, apoptotic and Wnt/β-Catenin signaling pathways, and inflammatory cytokines in their effects. Methods: The study included 90 male Sprague Dawley rats that were allocated to five groups (*n* = 18 rats): (I) Sham-operated group (right nephrectomy was performed); (II) Ischemia/reperfusion injury (IRI) group, a sham group with 45-min renal ischemia on the left kidney; (III) ATRA group, an ischemic group with an intravenous (i.v.) administration of ATRA 10 µM, 10 min post-surgery); (IV) WJ-MSCs group, an IRI group with an i.v. administration of 150 µL containing 7 × 10^6^ WJ-MSCs, 10 min post-surgery; (V) WJ-MSCs + ATRA group, an IRI group with an i.v. administration of 150 µL of 7 × 10^6^ WJ-MSCs pretreated with 10 µM ATRA. At the end of the experiments, serum creatinine, BUN micro-albuminuria (MAU), urinary protein, markers of redox state in the left kidney (MDA, CAT, SOD, and GSH), and the expression of Bax, IL-6, HIF-1α, Wnt7B, and β-catenin genes at the level of mRNA as well as for immunohistochemistry for NFkB and β-Catenin markers were analyzed. Results: The current study found that 45-min of renal ischemia resulted in significant impairment of kidney function (evidenced by the increase in serum creatinine, BUN, and urinary proteins) and deterioration of the kidney morphology, which was associated with a significant increase in redox state (evidenced by an increase in MDA and a decrease in GSH, SOD, and CAT), and a significant increase in inflammatory and apoptotic processes (evidenced by an increase in Bax and IL-6, NFkB, Wnt7B, β-catenin and HIF-1α) in kidney tissues (*p* < 0.05). On the other hand, treatment with ATRA, WJ-MSCs, or a combination of both, caused significant improvement in kidney function and morphology, which was associated with significant attenuation of oxidative stress, apoptotic markers, and inflammatory cytokines (IL6 and NFkB) with the upregulation of HIF-1α and β-catenin in kidney tissues (*p* < 0.05). Moreover, the renoprotective effect of WJ-MSCs pretreated with ATRA was more potent than WJ-MSCs alone. Conclusions: It is concluded that preconditioning of WJ-MSCs with ATRA may enhance their renoprotective effect. This effect could be due to the upregulation of the beta-catenin/Wnt pathway and attenuation of apoptosis, inflammation, and oxidative stress.

## 1. Introduction

Renal ischemia/reperfusion injury (IRI) is the most common cause of acute kidney injury (AKI) that results from impaired delivery of oxygen and nutrients to the kidney [1,2]. Renal IRI injury can occur in numerous clinical conditions; for example, partial nephrectomy, systemic hypo-perfusion with subsequent circulatory resuscitations, nucleation of renal cell carcinoma, renal vascular surgery, and local renal hypo-perfusion following renal transplantation [3]. The pathophysiology of renal IRI is still unclear; however, it is attributed to the depletion of ATP due to mitochondrial dysfunction [4]. It is also attributed to the activation of several enzymes, such as proteases, nitric oxide synthetases, and phospholipases that can disrupt cytoskeleton structure, which results in severe damage of the epithelial transporting mechanisms, and upregulation of inflammatory genes such as IL-6 and NFkβ, apoptotic genes such as Bax and genes of proliferative markers such as Wnt7β-β-catenin signaling pathways [4,5,6,7]. Although several studies have tried to understand the mechanisms underlying the pathophysiology of renal IRI, it is still a significant health issue.

The use of mesenchymal stromal/stem cells (MSCs) in the management of acute kidney injury (AKI) appears to be a promising therapeutic tool [8]. Wharton jelly-MSCs (WJ-MSCs) seem to have great potential in the treatment of kidney diseases due to the improvement of the glomerular filtration rate (GFR) and tubular function, downregulation of CD68-positive cell count and nuclear factor-kB (NFkB), upregulation of endothelial nitric oxide synthase (eNOS), vascular endothelial growth factor and Klotho genes that are associated with fibroblast growth factor expression, and attenuation of renal apoptosis [9]. Moreover, the classical effects of WJ-MSCs include their anti-inflammatory effects, pro-proliferative effects and anti-apoptosis effects through paracrine secretions [10]. However, there are several limitations to the use of MSCs in treating kidney disease, including its timing of administration and homing in the kidney tissues. It has been recommended that administration of MSCs should be within one hour after ischemic injury due to the lack of inflammation in the early injured kidney, which favors the survival of stem cells and helps the expression of homing adhesion molecules such as ICAM-1 and VCAM-1 that promote its homing into injured kidney tissues [11].

It has been demonstrated that retinoic acid (RA) is critical in kidney functions and development. It has been reported that the dietary lack of retinol during pregnancy results in inborn nephron deficits in rats [12]. It has also been demonstrated that RA is involved in the vascular remodeling in experimental nephritis and hypertrophy in uninephrectomy animal models [12]. Furthermore, lower than average concentrations of blood retinol were found in human acute renal failure (ARF) [13]. Trans-retinoic acid (ATRA) is considered the most common differentiating factor in a clinical trial [14]. Previous studies have reported that ATRA could differentiate hematopoietic and non-hematopoietic stem cells, e.g., hepatic progenitor cells, into hepatocyte-like cells [15,16]. We hypothesized that the pretreatment of WJ-MSCs with ATRA could potentiate or enhance the reno-treatment effects of WJ-MSCs against renal IRI by helping homing and differentiation of MSCs in kidney tissues. Therefore, the current study examined the effect of ATRA in the differentiation and homing of WJ-MSCs in a rat model of renal IRI and the possible role of oxidative stress, inflammatory cytokines, apoptotic genes, and differentiating cytokines in this process.

## 2. Materials and Methods

### 2.1. Experimental Animals

Ninety male Sprague Dawley rats weighing 200–300 g and aged 4–6 months were used in this study. They were bred and housed at Mansoura University’s Urology and Nephrology Center (UNC), animal research facility, where they were kept on a 12-h light-dark cycle. Animals were given a standard diet and ad libitum water. All experiments followed the NIK Guide for the Care and Use of Laboratory Animals and were approved by Mansoura University’s Faculty of Medicine’s IRB ethical committee (code# R.20.09.1011).

### 2.2. Isolation and Characterization of Wharton Jelly Stem Cells (WJ-MSCs)

All tissue samples were collected after written consent from mothers at the department of obstetrics, Mansoura University Hospital (MUH). After the caesarian section, about 15–20 cm of the umbilical cord was rapidly collected, rinsed in normal saline, and placed in a sterile container with medium (DMEM), penicillin, and streptomycin, then transported to the research facility at Mansoura UNC. The details of all steps of collecting WJ-MSCs from the human umbilical cord were described in a previous work by Yin et al. [17]. In brief, WJ was collected in a sterile centrifuge tube (falcon 50 mL), rinsed by PBS 7.4 solution, then isolated using 10 mL of collagenase type I and 5 mL of trypsin/EDTA, and centrifuged two times for 10 min at 800× *g* force. Then, the supernatant was removed, and the pelt was dissolved in 5 mL of complete medium. Then, the solution was transferred to a 25 cm flask and incubated in 5% CO_2_ at 37 °C. Finally, WJ-MSCs were characterized using a FACS caliber flow cytometer (Beckman, Fullerton, CA, USA) at the children’s hospital at Mansoura University for CD90, CD73, CD105, CD14, CD34, and CD45.

### 2.3. Cell Viability Assay

Cell viability was determined using the colorimetric MTT test. The cells were seeded at a density of 10 × 10^3^ cells per well in 96-well plates, then incubated for 24 and 48 h with varying concentrations of ATRA (0, 5, 7.5, 10 and 15 µM). The tissue culture was then treated for 4 h at 37 °C with 3-(4,5-Dimethylthiazol-2-yl)-2,5-diphenyltetrazolium bromide (Sigma Aldrich, St. Louis, MO, USA). The medium was discarded and 100 µL of DMSO (Sigma-Aldrich) was added and incubated for 10 min before measuring the optical density of solubilized formazan at 570 nm with an automatic microplate reader.

### 2.4. Labeling of WJ-MSCs before Transplantation

About 100 mg BrdU (5-Bromo-2′-Deoxyuridine) (Invitrogen, Thermo Fisher Scientific, Waltham, MA, USA) were dissolved in 32.5 mL anhydrous DMSO (Sigma-Aldrich, Burlington, MA, USA) to prepare a 10 µM stock solution. Then 10 µL of stock solution was diluted in 10 mL of 37 °C tissue culture medium to make a 10 µM labeling solution. The cells were trypsinized, counted, and collected in a sterile tube. Then, the cells were removed from the culture medium, placed in a BrdU labeling solution, and incubated at 37 °C for 2 h. The labeling solution was removed and the cells were washed two times with PBS. Finally, the cells were washed twice with a complete growth medium to be ready for transplantation [18].

### 2.5. Study Design

Rats were allocated to five groups (*n* = 18 rats) as follows: (I) Sham group: Rats underwent right nephrectomy with the exposure of the left kidney without clamping its pedicle; (II) IRI group, a sham group with the left renal pedicle clamping for 45 min [3]; (III) ATRA group, an IRI group with intravenous injection of 10 µM ATRA in 150 µL of complete growth medium after the release of the vascular clamp after 10 min [19]; (IV) WJ-MSCs group, an IRI group II with intravenous administration of 7 × 10⁶ WJ-MSCs suspended in 150 µL complete medium via the penile vein following vascular clamp release after 10 min [20]; (V) WJ-MSCs + ATRA group (*n* = 18), an IRI injury was induced similar to group II followed by intravenous administration of 150 µL of 7 × 10⁶ WJ-MSCs pretreated with 10 µM ATRA) [20]. Each group was subdivided into three subgroups, each with six rats, according to the time of sacrifice: three days, five days, and seven days after the surgery.

### 2.6. Collection of Blood Samples and Measurement of Serum Creatinine (SCr) and Blood Nitrogen Urea (BUN)

Blood samples were collected from the ophthalmic plexus (by very fine pasture petite under light halothane anesthesia at the end of the experiment for each subgroup) into a vacuum tube, then centrifuged to obtain the serum. Measurement of serum creatinine and blood nitrogen urea was carried out using commercially available colorimetric kits (Spinreact, Santa Coloma, Spain).

### 2.7. Collection of Urine Samples and Measurement of Urinary Protein Excretion

Urine samples were collected for 24 h in metabolism cages for each rat. The urine volume was calculated for 24 h, and urinary protein excretion was measured using commercially available colorimetric kits (Spinreact, Santa Coloma, Spain).

### 2.8. Collection of Kidney Specimens

At the end of the experiment, each animal was anesthetized by an overdose of Ketamine 300–360 mg/kg + xylazine 30–40 mg/kg combination intraperitoneally (i.p.). The abdomen was then opened again, and the kidney was quickly removed and bisected into two halves with a scalpel, with one half being quickly placed in a container containing 10% neutral buffered formalin for histopathological examination. The other half was quickly stored in liquid nitrogen in cryo-tubes for analysis of oxidative stress markers.

### 2.9. Measurement of MDA, SOD, CAT, and GSH in Kidney Tissues

Using a mortar and pestle, approximately 50–100 mg of kidney tissues were homogenized in 1–2 mL ice-cold buffer (50 mM potassium phosphate, pH 7.5, 1 mM EDTA), then centrifuged at 1400× *g* force for 15 min at 4 °C. The supernatant was stored at −20 °C until it was used for oxidant and antioxidant analyses. Measurements of renal cortical malondialdehyde (MDA) (as a lipid peroxidation index) and superoxide dismutase (SOD) (an endogenous antioxidant enzyme), catalase (CAT), and reduced glutathione (GSH) were used to estimate renal oxidative stress following I/R injury. According to the manufacturer’s instructions, these markers were quantified using a colorimetric method in the supernatant of kidney homogenates (Bio-Diagnostics, Dokki, Giza, Egypt).

### 2.10. Assessment of mRNA Levels of Genes Expression in Kidney Tissue by Real-Time qPCR

Total RNA was isolated from the kidney tissue specimens by immersing 50–100 mg of tissue in 1 mL of TRIzolTM (Invitrogen, USA). After being quantified spectrophotometrically, the RNA integrity was assessed using agarose gel electrophoresis and ethidium bromide staining. For real-time RT-PCR, only samples of acceptable quality that showed clear 18S and 28S bands under ultraviolet light were used. One µg total RNA and a cDNA synthesis kit were used for reverse transcription (high-capacity cDNA archive kit) (Applied Biosystems™, Waltham, MA, USA). The primer sequences for the tested genes were conducted commercially (Vivatntis, Oceanside, CA, USA) in the following sequences: **GAPDH** F: 5′-AGACAGCCGCATCTTCTTGT-3′ R: 5′-TTCCCATTCTCAGCCTTGAC-3′, **Wnt7β** F: 5′-CCATCATCGTGATCGGGGAG -3′ R: 5′-CAAAGTAGAAAAGGGCAACC -3′, **HIF-1α** F: 5′-TGCTTGGTGCTGATTTGTGA-3′ R: 5′-GGTCAGATGATCAGAGTCCA-3′, **β-catenin** F: 5′-TGAAGGTGCTGTCTGTCTGCTC-3′ R: 5′-TGCATCGGACAAGTTTCTCAGA-3′, **Bax** F: 5′-GGCGATGAACTGGACAACAA-3′ R: 5′-CAAAGTAGAAAAGGGCAACC-3′, **IL-6** F: 5′-GCCCTTCAGGAACAGCTATGA-3′ R: 5′TGTCAACAACATCAGTCCCAAGA 3′.

### 2.11. Real-Time PCR Reaction

In a total volume of 50 µL, the reaction was carried out with 25 µL of TaqMan^®^ Universal PCR (Applied Biosystems^™^, USA), 2.5 µL of 209 TaqMan^®^ Gene Expression Assay Mix, and 22.5 µL of cDNA diluted in RNase-free water. Initial denaturation at 95 °C for 10 min was followed by 40 cycles of denaturation at 95 °C for 15 s, annealing at 60 °C for 1 min, and extension at 72 °C for 1 min. Equation 2^−ΔΔCT^ was used to analyze the data using ABI prism 7000.

### 2.12. Histopathological Examination of Kidney Tissues

The kidney specimens were prepared for paraffin blocks, and 3-μm thick slices were prepared and stained with hematoxylin and eosin before being examined under a light microscope. Tubular dilatation, distal tubular cast, proximal tubular brush border loss, patchy loss of tubular cells, peritubular vascular congestion, endothelial damage, and leukocyte accumulation were investigated in tubulointerstitial regions. A well-defined scoring system was used to evaluate the degree of active injury changes, tubulointerstitial damage, and degenerative changes. The tubulointerstitial damage score included inflammatory cells infiltrating the interstitial space and necrotic tubules. Necrotic tubules were scored based on the number of necrotic tubules counted per high power field (HPF). They were given a score of 1, 2, 3, or 4 for 0, 1–3, 4–5, and >5 necrotic tubules per HPF, respectively. Mild, moderate, and severe grades of inflammatory cells were scored on a scale of 1, 2, or 3. An active injury had a maximum score of 5. Mitosis, solid cellular sheets between tubules, intraluminal cellular proliferation generating solid tubules, tubules lined with big vesicular nuclei, and tubules lined by cells with prominent hyperchromatic nuclei and scant cytoplasm all contributed to the luminal border’s festooned appearance.

### 2.13. Immunohistochemistry Investigation for Expression of NFkB and β-Catenin in Kidney Tissues

Formalin-fixed paraffin sections (3 µm) were used for the assessment of apoptotic marker (NF-κB) and proliferative marker β-catenin by immunohistochemical examination according to a previously published guideline [21,22]. Briefly, fixed sections were deparaffinized in xylene and hydrated in graded ethanol (100%, 95%, and 80%) for 1 min then rinsed with water, and the slides were treated with 10 mM Citrate Buffer pH 6.0 as antigen retrieved for 15 min in an oven then cooled for 20 min and washed. Then, 3% H_2_O_2_ was used for 10 min to block endogenous peroxidase activity and the slides were washed with distilled H_2_O. The slides were incubated overnight with primary antibodies NFkB and beta-catenin at a dilution of 1:100 at 4 °C and then washed with PBS. The slides were incubated with poly HRP conjugate for 30 min at room temperature and then washed with PBS. After that, slides were incubated with DAB chromogen monitoring staining development. They were washed, counterstained with hematoxylin, dehydrated, cleared, and coverslipped for picturing using an Olympus light microscope. The expression was quantified by calculating the number of positive cells in five high power fields (HPF), and the average of the fields was estimated.

### 2.14. Statistical Analysis

The statistical analysis was performed by SPSS software. A paired T-test was used to compare two quantitative variables. A two-way ANOVA version 2.3 with Tukey’s post-hoc test was used to compare more than two quantitative variables. Histopathological analyses were analyzed by Kruskal–Wallis followed by Mann–Whitney’s tests; differences were statistically significant at *p* < 0.05.

## 3. Results

### 3.1. Tissue Culture and WJ-MSCs Characterizations

**Figure 1:**
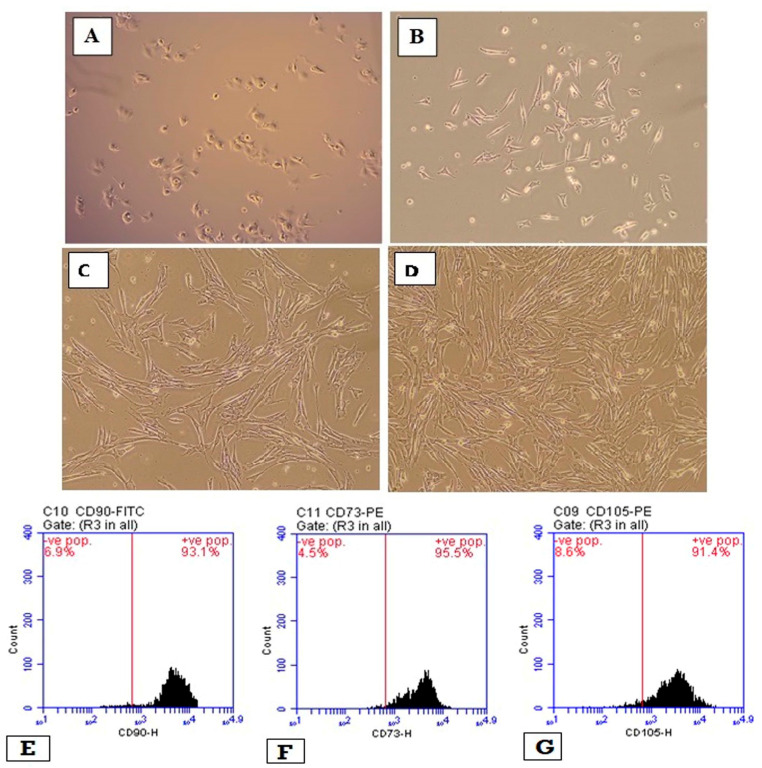

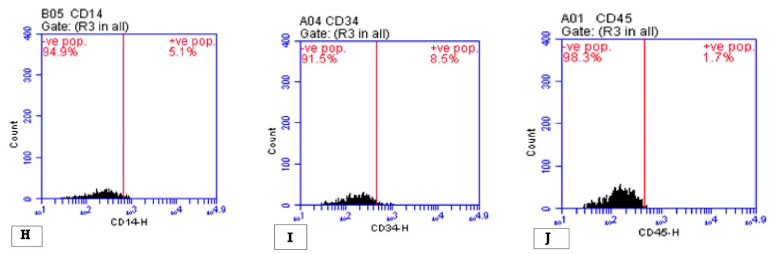
WJ-MSCs in tissue cultures and their characterization. (**A**) shows images of WJ-MSCs alone on day 1 (100× magnification), while (**B**) shows images of WJ-MSCs alone at passage 3 (100× magnification). (**C**) shows WJ-MSCs which have a mesenchymal-like shape with a flat polygonal morphology (100× magnification), and (**D**) shows WJ-MSCs treated with 10 µM ATRA with 80% confluence (100× magnification). Flow cytometry histograms for WJ-MSCs showing positivity for CD90 (**E**), CD73 (**F**), CD105 (**G**) and negativity for CD 14 (**H**), CD34 (**I**) and CD45 (**J**). WJ-MSCs = Wharton jelly-mesenchymal/stromal stem cells.

Figure 1A–D shows spindle-shaped cells in tissue culture after three days. After 14 days, the spindle-shaped cells reached 80% confluence. After the third passage, cultures were composed of a homogenously fibroblastic cell monolayer. In addition, WJ-MSCs were negative to markers CD14, CD34 and CD45 with percentages of 96.9%, 94.5% and 97.0%, respectively (Figure 1E–G) and positive for CD90, CD73 and CD105 with percentages of 82.8, 72.4% and 84.9%, respectively (Figure 1H–J).

### 3.2. Cell Viability Assay and Homing of WJ-MSCs with BrdU in Kidney Tissues

**Figure 2:**
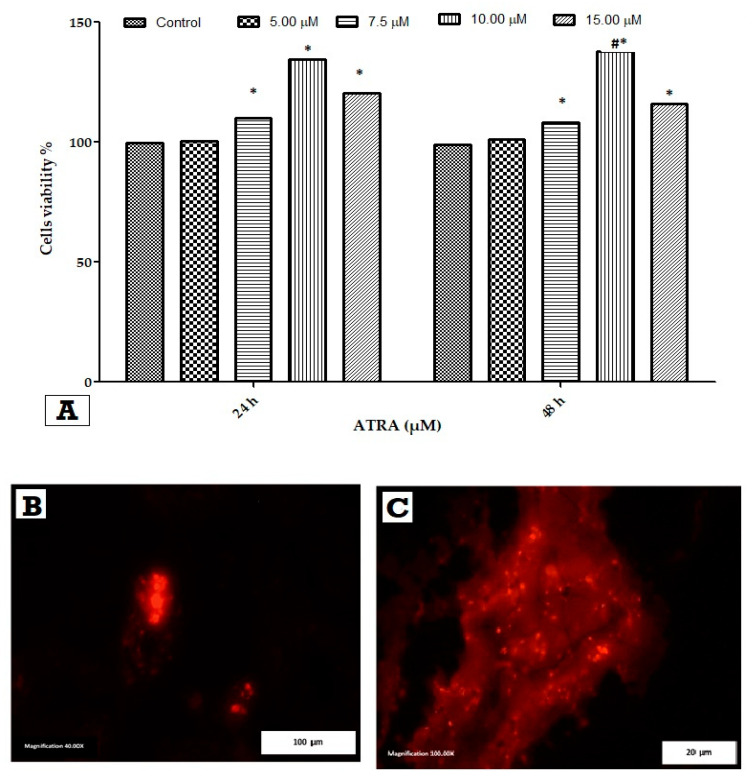
WJ-MSCs viability and homing. (**A**) shows the effect of ATRA on WJ-MSCs viability, * compared with control, # compared with 10 µM ATRA at 24 h. Homing of labelled WJ-MSCs with BrdU in kidney tissue at 100 µm scale bar and magnification 400×, and in (**B**) at 20 μm scale bar and magnification at 100×. (**C**) shows the enlargement and irregularity of BrdU-labelled capsular nuclei (arrow). Representative microphotographs of a kidney section were obtained 3 days after ischemia followed by intravenous administration of 7 × 10⁶ MSCs by 10 min.

The viability of WJ-MSCs exposed to various doses of ATRA was assessed using the MTT assay for 24 and 48 h. ATRA significantly increased WJMSC proliferation at 24 and 48 h (Figure 2A). Except for 5 μM ATRA, the viability of WJ-MSCs was significantly greater in all treated WJ-MSCs for 24 and 48 h compared with the control (*p* ≤ 0.05). After a concentration of 10 μM, viability increased the most at 48 h compared with 24 h and other treated WJ-MSCs (*p* ≤ 0.05). Additionally, WJ-MSCs with BrdU labeling was deposited in the kidney and scattered in the glomeruli, as shown in Figure 2B,C, which shows BrdU-positive cells (red) expressed after injury.

### 3.3. Serum Creatinine (SCr) and Blood Nitrogen Urea (BUN), and Urinary Protein Excretions

Basal values of serum creatinine were comparable among different subgroups. Test values of serum creatinine in IRI, ATRA, and WJ-MSCs groups were significantly higher than the sham group on days 3, 5, and 7 (*p* ≤ 0.05). On day 7, compared with the sham group, IRI and ATRA groups showed significantly higher serum creatinine values, and the WJ-MSCs group showed statistically lower values (*p* ≤ 0.05). On the other hand, the ATRA+ WJ-MSCs group showed statistically significant lower values of serum creatinine compared with IRI, ATRA, and WJ-MSCs groups on days 3, 5, and 7 (*p* ≤ 0.05) (Table 1).

In addition, basal values of serum BUN were comparable among different subgroups. Test values of serum BUN in IRI, ATRA, and WJ-MSCs groups were significantly higher than the sham group on days 3, 5, and 7 (*p* ≤ 0.05). On the other hand, all treated groups (ATRA, WJ-MSCs, and ATRA + WJ-MSCs) showed statistically significant lower values compared with the IRI group (*p* ≤ 0.05). Moreover, the ATRA + WJ-MSCs group showed statistically significant lower values of serum BUN compared with the IRI, ATRA, and WJ-MSCs groups on days 3, 5, and 7 (*p* ≤ 0.05) (Table 1).

Furthermore, basal values of urinary protein excretion were comparable among different subgroups. Urinary protein test values in IRI groups were significantly higher than in the sham group on days 3, 5, and 7 (*p* ≤ 0.05). In contrast, all treated groups (ATRA, WJ-MSCs, and ATRA + WJ-MSCs) showed statistically significant lower values compared with the IRI group (*p* ≤ 0.05). In addition, the ATRA + WJ-MSCs group showed statistically significant lower values of urinary proteins compared with the IRI and ATRA groups on day 3 (*p* ≤ 0.05) (Table 1).

### 3.4. The Concentrations of MDA and GSH and Activities of SOD and CAT in Kidney Tissues

Compared with the sham group, the IRI group demonstrated a significant increase in MDA levels in kidney tissues on days 3, 5, and 7 (*p* ≤ 0.05). On the other hand, all treated groups (ATRA, WJ-MSCs, and ATRA + WJ-MSCs) showed statistically significant lower values compared with the IRI group (*p* ≤ 0.05). Moreover, the ATRA + WJ-MSCs group showed a statistically significantly lower MDA level in kidney tissues than IRI (*p* ≤ 0.05). Additionally, the ATRA + WJ-MSCs groups showed a significant decrease in MDA levels in kidney tissues compared with the ATRA group and WJ-MSCs group (*p* ≤ 0.05) at different intervals (Table 2).

On the other hand, the IRI group showed a significantly lower concentration of GSH and activities of SOD and CAT in kidney tissues on days 3, 5, and 7 than the sham group (*p* ≤ 0.05). However, all treated groups (ATRA, WJ-MSCs, and ATRA + WJ-MSCs) showed statistically significantly higher values for these parameters compared with the IRI group (*p* ≤ 0.05). Moreover, ATRA + WJ-MSCs groups showed significantly higher values of these parameters in the kidney tissues compared with the ATRA and WJ-MSCs groups (*p* ≤ 0.05) at different intervals (Table 2).

### 3.5. Expression of IL6, HIF-1α, Bax and Wnt7β Genes at the Level of mRNA

**Figure 3:**
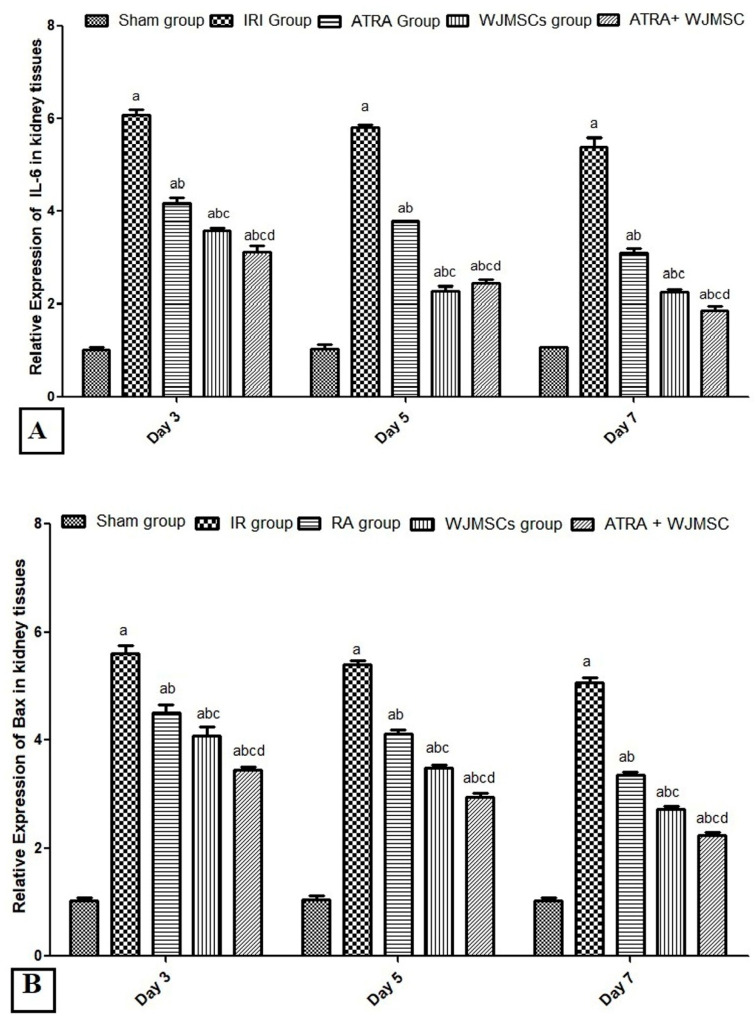

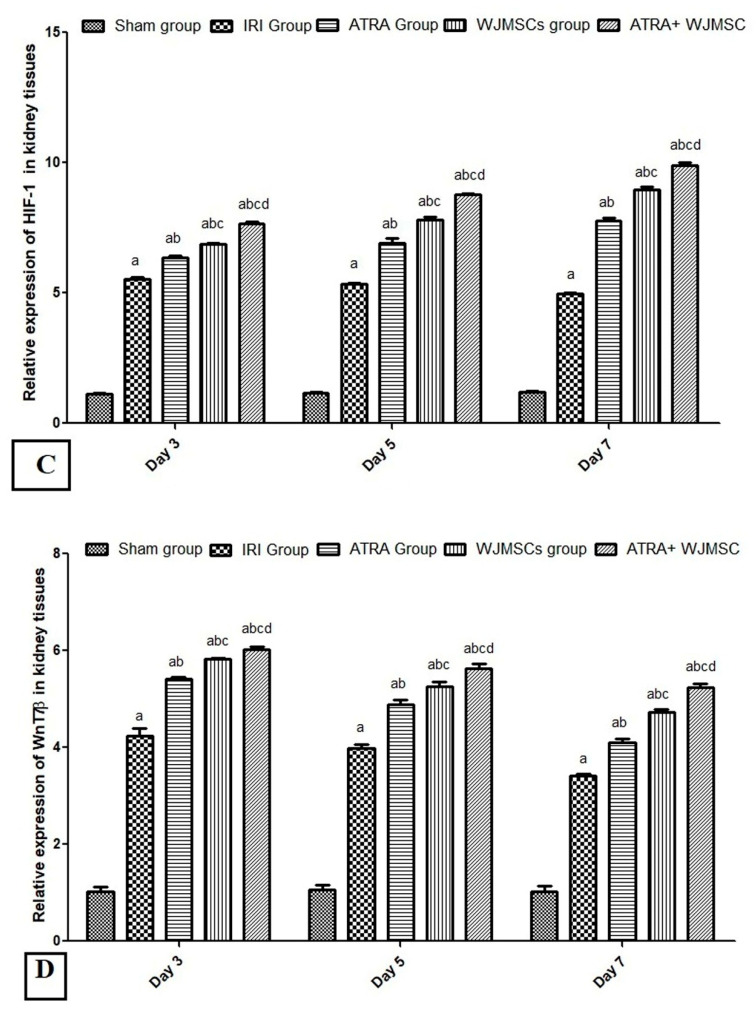

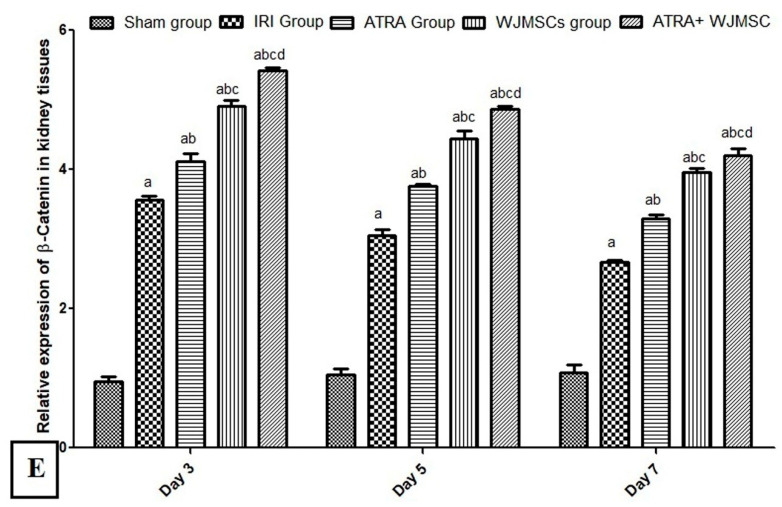
Effect of ATRA and WJ-MSCs on the expression of IL6 (**A**), Bax (**B**), HIF-1α (**C**), WnT7B (**D**) and β-catenin (**E**) genes at the level of mRNA in kidney tissues (*n* = 6/group). The values are mean ± SD for six rats per group. One-way ANOVA test with post-hoc Scheffe’s test (significant if *p* ≤ 0.05). a Significant vs. sham group of the same time interval; b significant vs. IR group of the same time interval; c Significant vs. ATRA group of the same time interval; d Significant vs. WJ-MSCs group of the same time interval.

Compared with the sham group, the IRI group showed significantly higher expression for IL-6 and Bax genes in the kidney tissues on days 3, 5, and 7 (*p* ≤ 0.05). On the other hand, all treated groups (ATRA, WJ-MSCs, and ATRA + WJ-MSCs) showed a significant reduction in the expression of these genes compared to the IRI group (*p* ≤ 0.05). Moreover, the ATRA + WJ-MSCs group showed a significant decrease in the expression of IL6 and Bax genes in kidney tissues compared with the ATRA and WJ-MSCs groups (*p* ≤ 0.05) at different intervals (Figure 3A,B).

The expressions of HIF-1alpha, Wnt7beta, and beta-catenin were significantly increased in kidney tissues of the IRI group compared with the sham group on days 3, 5, and 7 (*p* ≤ 0.05). However, all treated groups (ATRA, WJ-MSCs, and ATRA + WJ-MSCs) showed statistically significant higher values of these tested genes compared with the IRI group (*p* ≤ 0.05). Moreover, the ATRA + WJ-MSCs group showed a significant increase in HIF1α, Wnt7beta, and beta-catenin expressions in kidney tissues compared with the ATRA and WJ-MSCs groups (*p* ≤ 0.05) at different intervals (Figure 3C–E).

### 3.6. Histopathological Studies for Kidney Tissues (H&E)

**Figure 4:**
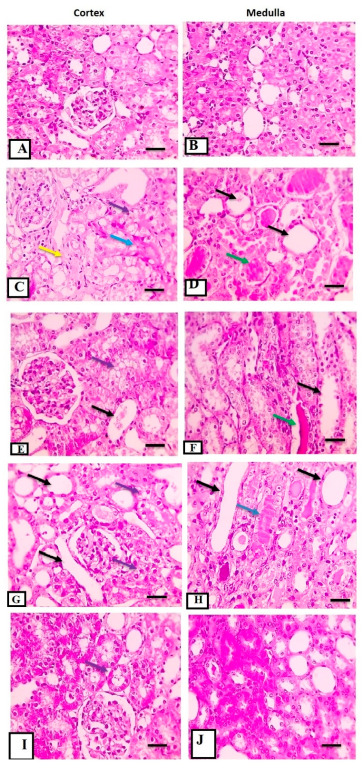
Microscopic pictures of H&E-stained renal sections from rats for treatment groups (i) to (v). (i) The sham group on day 7 shows the normal structure of glomeruli, PCT, and DCT in the cortex (**A**) and normal tubules in the medulla (**B**) (high magnification 400×, scale bar 50 µm). (ii) The IRI group shows dilated irregular tubules with attenuated epithelial lining (black arrows) with apoptotic cells (red arrows), tubular hyaline casts (green arrows), degenerated (violet arrows) and necrotic (blue arrows) epithelial lining, interstitial fibrosis infiltrated with inflammatory cells mainly lymphocytes (yellow arrows) in the cortex (**C**) and medulla (**D**) (high magnification 400×, scale bar 50 µm). (iii) The ATRA group shows a decrease in severe tubular dilation (black arrows), degeneration (violet arrows) and cast formation in the cortex (**E**) and medulla (**F**) (high magnification 400×, scale bar 50 µm). (iv) The WJ-MSCs group shows milder lesions than recorded in the IRI group and ATRA group characterized by mild tubular dilation and degeneration (violet arrows) in the cortex (**G**) and medulla (**H**), infrequent cast formation and few apoptotic cells (high magnification 400×, scale bar 50 µm). (v) The ATRA + WJ-MSCs group shows much milder lesions than recorded in previous groups characterized by very mild tubular dilation and degeneration (violet arrows) in the cortex (**I**) and medulla (**J**) and infrequent cast formation in the medulla (high magnification 400×, scale bar 50 µm).

The IRI group showed a significant increase in tubulointerstitial damage scores (in the form of tubular dilatation, casts, loss of brush border, interstitial hemorrhage, and inflammatory cell infiltrations) in both the cortex and medulla on days 3, 5, and 7 (*p* ≤ 0.05). On the other hand, all treated groups (ATRA, WJ-MSCs, and ATRA + WJ-MSCs) showed a statistically significant decrease in tubulointerstitial damage scores and active injury changes in both the cortex and medulla compared with the IRI group (*p* ≤ 0.05). Moreover, the ATRA + WJ-MSCs groups showed a significant decrease in tubulointerstitial damage scores and active injury changes in both the cortex and medulla in the kidney tissues compared with the ATRA and WJ-MSCs groups (*p* ≤ 0.05) at different interval times (Table 3 and Figure 4A–E).

Regeneration scores for the IRI groups showed non-significant changes compared with the sham group on days 3, 5, and 7 (*p* > 0.05). In contrast, all treated groups (ATRA, WJ-MSCs, and ATRA + WJ-MSCs) showed a statistically significant increase in regeneration scores in both the cortex and medulla compared with the IRI group (*p* ≤ 0.05). Moreover, the ATRA + WJ-MSCs groups showed a significant increase in TID and active injury changes in both the cortex and medulla in the kidney tissues compared with the ATRA and WJ-MSCs groups (*p* ≤ 0.05) at different interval times (Table 3 and Figure 4A–E).

### 3.7. Expression of β-Catenin & NF-κβ in Kidney Tissues by Immunohistochemistry

**Figure 5:**
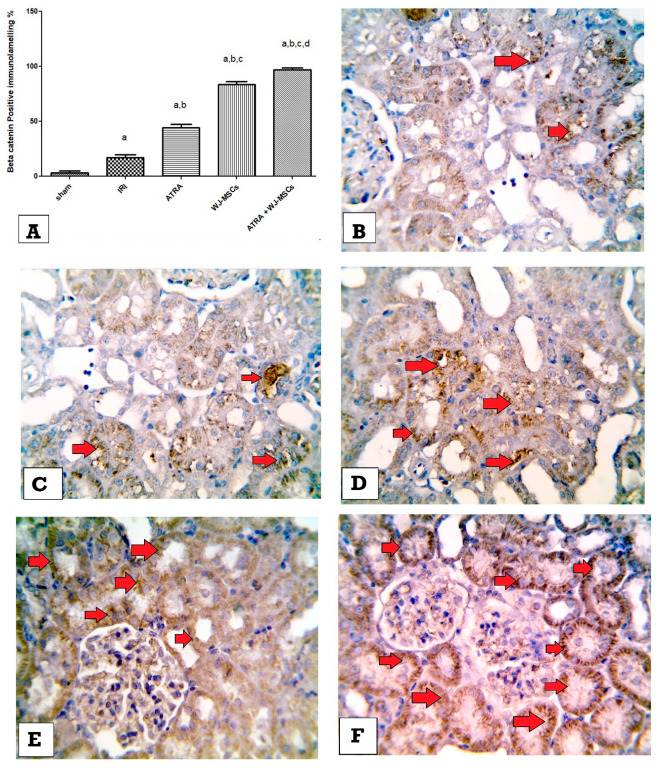
Expression of β-catenin in kidney tissues in day 5 group. (**A**) Percentage of β-catenin positive cells in different studied groups. ^a^ significant vs. sham group, ^b^ significant vs. IRI group, ^c^ significant vs. ATRA group, ^d^ significant vs. WJ-MSCs group. Microscopic pictures of immunostained tissues showing very minimal brown expression of β-catenin in renal tubular epithelial cells and glomerular cells of sham group (red arrows) (**B**), mild brown expression of β-catenin in renal tubules of the IRI control group (red arrows) (**C**), mild to moderate expression of β-catenin in renal tubules of ATRA group (red arrows) (**D**), moderate expression of β-catenin in renal tubules of WJ-MSCs group (red arrows) (**E**), and high expression of β-catenin in renal tubules of the ATRA + WJ-MSCs group (red arrows) (**F**) (high magnification 400×).

Immunohistochemical examination revealed a significant increase in beta-catenin expression in kidney tissues of the IRI group compared with the sham group (*p* ≤ 0.05). On the other hand, β-catenin expression levels were significantly increased in all treated groups compared with the IRI group (*p* ≤ 0.05) and were the most significant in the ATRA + WJ-MSCs group compared with the IRI control group on day 5. Moreover, the ATRA + WJ-MSCs group showed a significant increase in β-catenin compared with the ATRA and WJ-MSCs groups (*p* ≤ 0.05) on day 5 (Figure 5A–F).

**Figure 6:**
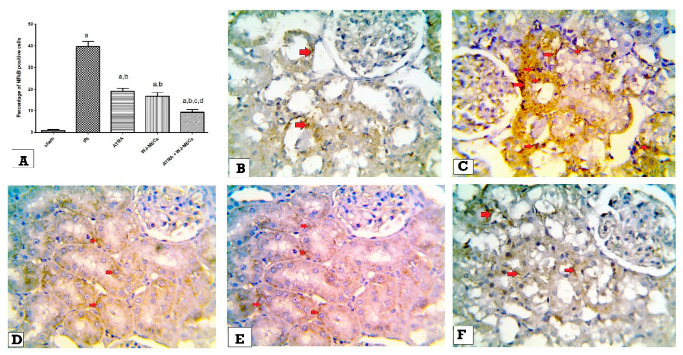
Expression of NFkB in kidney tissues in day 5 group. (**A**) Percentage of NFkB positive cells in different studied groups. ^a^ significant vs. sham group, ^b^ significant vs. IRI group, ^c^ significant vs. ATRA group, ^d^ significant vs. WJ-MSCs group. Microscopic pictures of immunostained tissues showing negative expression of NFkB in renal tubular epithelial cells and glomerular cells of sham group (red arrows) (**B**), high brown expression of NFkB in renal tubules of the IRI control group (red arrows) (**C**), moderate expression of NFkB in renal tubules of ATRA group (red arrows) (**D**), mild expression of NFkB in renal tubules of WJ-MSCs group (red arrows) (**E**), and low expression of NFkB in renal tubules of ATRA + WJ-MSCs group (red arrows) (**F**) (high magnification 400×).

Regarding NF-κB expression, the IRI group showed a significant increase in its expression compared with the sham group (*p* ≤ 0.05). In contrast, the expression of NF-κB was significantly reduced in all treated groups (*p* ≤ 0.05) to be the lowest in the ATRA + WJ-MSCs group compared with the IRI control group on day 5. Moreover, ATRA + WJ-MSCs groups demonstrated a significant decrease compared with the ATRA and WJ-MSCs groups (*p* ≤ 0.05) on day 5 (Figure 6A–F).

## 4. Discussion

The recovery process of renal cells requires the replacement or regeneration of the damaged cells and prevention of apoptosis, inflammation, and oxidative stress [23,24,25]. In the current study, we adopted a well-established model of renal ischemia (45 min left renal ischemia and contralateral right nephrectomy) to examine the pretreatment of WJ-MSCs with ATRA on the recovery of the kidney tissues after the ischemic injury. The current study showed that renal ischemia resulted in significant impairment in renal glomerular and tubular functions, as evidenced by a significant rise in serum creatinine, BUN, and urinary proteins at various follow-up intervals. These findings agree with those of previous studies [26,27,28] and show that IRI injury resulted in glomerular and tubular dysfunctions. In addition, morphological examination revealed that the kidneys of the ischemic group had more renal tubular necrosis, loss of brush borders, intra-tubular casts, and interstitial inflammatory cells, all of which explained the impairment of the glomerular and tubular functions of the kidneys and confirmed the development of acute tubular necrosis, which is a pathognomonic feature of renal IRI. We also found moderate regenerative capacity in the kidney tissues, particularly within 7 days, in the form of mitotic figures, prominent nucleoli, and solid masses of sheets. These findings were in line with previous studies [3,26].

The first objective was to examine the effect of ATRA, WJ-MSCs, and WJ-MSCs pretreated with ATRA on renal IRI. The present study demonstrated that treatment with 10 um ATRA alone did not cause a significant improvement in the markers of kidney functions, including serum creatinine, BUN and urinary proteins with minor morphological changes in the kidney’s degenerative and regenerative capacities. To the best of our knowledge, these are the first reported findings regarding the effect of ATRA alone in the case of renal ischemia, suggesting that exogenous ATRA has less effect on kidney function and morphology in renal ischemia than these findings of WJ-MSCs treated with ATRA. Furthermore, we reported significant improvement in renal function parameters, including serum creatinine, BUN, urinary proteins, and kidney morphology in the WJ-MSCs group, suggesting and confirming the renoprotective effect of the WJ-MSCs on renal IRI injury. These findings agreed with previous experimental studies that demonstrated the effectiveness of using stem cell therapy against renal IRI injury [9,29,30,31].

Moreover, the present study demonstrated a significant improvement in markers of kidney functions, including serum creatinine, BUN, and urinary proteins in WJ-MSCs pretreated with 10 µM ATRA more than WJ-MSCs that were not pretreated with it. The dose of 10 um ATRA was chosen in the present work because this dose exhibited a powerful enhancing effect on the cell viability of WJ-MSCs. In line with these findings, the histopathological examination of the kidney tissues showed significant attenuation in acute tubular necrosis scores with a significant increase in the regenerative capacity of renal tubules other than WJ-MSCs alone. These findings suggest that the renoprotective effect of this combination, and ATRA might improve the homing of WJ-MSCs in the injured tissues, enhancing its protective potency in the injured tissues. To the best of our knowledge, these findings were the first to demonstrate the potentiating effects of ATRA on WJ-MSCs against renal IRI injury. In line with these findings, Zhu et al. [32] also demonstrated that ATRA potentiated the repairing effects of stem cells on the myocardial ischemic injury.

The second objective of the current study was to investigate the possible underlying mechanisms for this potentiating effect of ATRA on the renoprotective effects of WJ-MSCs. We hypothesized that this could be attributed to attenuation of the injury process due to oxidative stress, apoptosis and inflammation, and enhancement of the process of regeneration of renal epithelial cells. The role of reactive oxygen species (ROS) in IRI has been investigated extensively, and they result in severe tissue injury, which is further induced via an inflammatory response [33,34]. The present study showed that the IRI group demonstrated elevated MDA and reduced renal antioxidants, including GSH concentration, and reduced activities of SOD and catalase, in line with previous studies [35,36]. On the other hand, treatment with either ATRA, WJ-MSCs, or WJ-MSCs pretreated with ATRA caused significant improvement in oxidative stress as evidenced by a significant reduction in MDA with a significant increase in GSH concentrations and SOD and catalase activities in all treated groups compared with the untreated ischemic group. Moreover, the antioxidant effect of the WJ-MSCs pretreated with ATRA was more potent than WJ-MSCs alone, suggesting that ATRA potentiates the antioxidant effect of WJ-MSCs in ischemic kidney tissues. Keshtkar et al. [37] found that WJ-MSCs protected the kidney against ischemia-reperfusion injury by reducing oxidative and endoplasmic reticulum stresses via activation of hypoxia-inducible factor-1 α (HIF-1α), so in the current study we investigated the effect of WJ-MSCs and its combination with ATRA on the expression of HIF-1α. HIF-1α plays a critical role in cellular oxygen homeostasis and mitochondrial respiration and protects the kidney against cisplatin-induced tubular cell apoptosis and oxidative stress. HIF-1α a subunit is an O2-regulated subunit induced by hypoxia, oxidative stress, and some transcription factors. During renal IRI, the enhanced oxidative stress causes a significant increase in HIF-1 α expression, as demonstrated in the current work and reported by previous studies [27,38]. Moreover, the current work demonstrated a significant increase in HIF-1α in stem cell-treated groups, and its expression became more marked in the ischemic group treated with WJ-MSCs pretreated with ATRA, suggesting that ATRA could enhance the antioxidant effects of WJ-MSCs via activation of HIF-1α.

It has been documented that renal IRI is a complex inflammatory process in which inflammation and inflammatory cytokines play a significant role in its pathophysiology. One of the inflammatory cytokines, involved in ischemic injury is IL-6, and its production is increased during ischemia in many organs such as the brain [39], gut [40], and heart [41], and the amount of IL-6, correlates with the amount of ischemic injury [42]. In agreement with previous studies, we found in the current study significant upregulation of IL-6 expression in ischemic kidneys [27,43]. In addition, we found significant downregulation of IL-6 in all treated groups, and the largest reduction in IL-6 expression was found in kidney tissues treated with WJ-MSCs pretreated with ATRA suggesting anti-inflammatory effects for WJ-MSCs. In agreement with the current study’s findings, Hussein et al. [27] reported anti-inflammatory activity for bone marrow-derived MSCs in renal IRI.

In addition, apoptosis is one of the critical features of apoptosis and involves activating pro-apoptotic proteins such as Bax and Bak. Bax is one of the principal executioners in renal tubular apoptosis, central to apoptotic pathways that implicate the mitochondria and promote the release of caspase activators, such as cytochrome c [44]. Another study added that Bax/Bcl2 mediated apoptosis is essential in the execution of apoptotic cell death in renal ischemia [45]. According to previous studies, our results also confirm a significant increase in Bax expression in kidney tissues of the untreated ischemic group. On the other hand, treatment with either WJ-MSCs or WJ-MSCs pretreated with ATRA caused a significant reduction in Bax expression, which was more potent in WJ-MSCs pretreated with the ATRA group, suggesting that the renoprotective effect for WJ-MSCs involves anti-apoptotic actions. In agreement with these findings, Xu et al. [46] demonstrated that human WJ-MSCs significantly improves the kidney function and morphology in cisplatin acute and chronic toxicity via reduction of collagen deposits, the ratio of Bax to Bcl-2, and transforming growth factor β mRNA expression, and prevents the epithelial-mesenchymal transition (EMT) in renal injury tissues. In addition, Condor et al. [9] showed that human WJ-MSCs protects the kidney in a rat model of sepsis by improving glomerular filtration rate, tubular function, decreased nuclear factor κB, and cytokine expression, increased expression of eNOS and Klotho, attenuated renal apoptosis, and improved survival.

We reason that because Wnt/-catenin signaling was required for primary nephrogenesis, it might also be required for IRI’s endogenous repair mechanism and regeneration process. Nonetheless, this signaling pathway is activated in kidney tubules, podocytes, and interstitial cells under numerous conditions, including diabetic nephropathy, cystic kidney disease, and acute kidney injury (AKI) [47]. Zhang et al. [48] found that the endogenous β-catenin/Wnt pathway is reactivated during AKI recovery and required for integrating exogenous embryonic renal progenitor cells into damaged tubules. Furthermore, recently Anderova et al. [49] reported that Wnt signaling hyper-activation increased the abundance of proliferating and neuron-like cells in focal cerebral ischemia. The current study demonstrates a minimal increase in the expression of Wnt7/β-catenin signaling expression in kidney tissues of the ischemic group. However, treatment with WJ-MSCs alone or WJ-MSCs or WJ-MSCs pretreated with ATRA upregulated β-catenin expression levels. Moreover, WJ-MSCs pretreated with ATRA caused a more significant increase in their expressions than WJ-MSCs alone. These findings suggest that the Wnt7/beta-catenin pathway might repair and recover renal epithelial cells during IRI by WJ-MSCs. In contrast with these findings, Jiao et al. [50] reported that the Wnt/β-catenin pathway is involved in repairing renal tubular cells following AKI. The results of the current study also suggest that pretreatment of WJ-MSCs with ATRA could enhance its effect on the activation of the Wnt/beta catenin pathway.

NF-κB (nuclear factor kappa-light-chain-enhancer of activated B cells) is a family of structurally related eukaryotic transcription factors that affect many cellular activities such as immunological responses, inflammation, apoptosis, growth, and development. Increased NF-κB expression has been observed in various disorders, indicating that genetic dysregulation plays a role in disease pathophysiology [51]. This study reported that NF-κB expression levels decreased in all treated groups to be the highest in the ATRA + WJMSC group. On the other hand, the ATRA + WJ-MSCs group demonstrated a significant decrease compared with the ATRA and WJ-MSCs groups. These results agree with those of Marko et al. [52], who studied tubular epithelial-specific NF-κB activation in a mouse model of ischemia-reperfusion injury (IRI)-induced AKI. IRI generated widespread NF-κB activation in renal tubular epithelial and interstitial cells, which peaked 2–3 days after injury, according to NF-κB reporter activity and nuclear localization of phosphorylated NF-κB subunit p65 studies in mice. Rap1 is vital in regulating MSC paracrine functions, according to Zhang et al. [48]. Rap1^−/−^-BM-MSCs lowers NF-κB sensitivity to stress-induced pro-inflammatory cytokine secretion and apoptosis to improve MSC-based therapeutic efficacy in myocardial infarction compared with BM-MSCs. Our results indicate that the most influential group is the ischemic treated with both ATRA and WJ-MSCs group, which proves the ability of ATRA to improve the functions of WJ-MSCs and agrees with Sabbaghziarani et al. [53]. They documented the potential of retinoic acid-pretreated WJ-MSCs in conjunction with triiodothyronine to increase neurotrophic factor expression in the subventricular zone of ischemic brain damage in rats. WJ-MSCs can differentiate into diverse cell types and stimulate tissue regeneration, according to a recent study [54]. However, WJMSC transplantation’s regeneration potential was limited by their poor differentiation and survival rate [55,56]. Thus, ATRA treatment might be a practical new approach to stimulate the differentiation of WJ-MSCs into hepatocyte-like cells [16].

## 5. Conclusions

The present study has confirmed that IRI has destructive effects on renal tissue and revealed that ATRA might be an essential indicator in cultured WJ-MSCs by inducing multiple chemokine receptors and angiogenic factors. ATRA reduces apoptosis and induces cell migration. These beneficial effects of ATRA administration may be related to its antioxidant and anti-inflammatory effects. Therefore, preconditioning of WJ-MSCs with ATRA before transplantation could enhance their therapeutic capacity.

## Figures and Tables

**Table 1:** Effect of pretreatment of WJMSCs with ATRA on serum creatinine (SCr), serum BUN and urinary protein excretion (g/24 h) at 3, 5 and 7 days (n = 6/group).

Serum Creatinine (mg/dL)						
**Groups**	**Day 3**		**Day 5**		**Day 7**	
	**Basal**	**Test**	**Basal**	**Test**	**Basal**	**Test**
**Sham**	0.4 ± 0.14	0.57 ± 0.12	0.48 ± 0.15	0.51 ± 0.1	0.41 ± 0.11	0.58 ± 0.14
**IRI**	0.41 ± 0.12	2.83 ± 0.51 ^a^	0.41 ± 0.14	2.24 ± 0.62 ^a^	0.4 ± 0.08	1.68 ± 0.29 ^a^
**ATRA**	0.41 ± 0.09	1.82 ± 0.33 ^a,b^	0.43 ± 0.1	1.25 ± 0.34 ^a,b^	0.41 ± 0.11	0.84 ± 0.19 ^b^
**WJ-MSCs**	0.45 ± 0.14	1.39 ± 0.21 ^a,b^	0.43 ± 0.12	1.14 ± 0.13 ^a,b^	0.38 ± 0.14	0.73 ± 0.12 ^b^
**ATRA + WJ-MSCs**	0.45 ± 0.13	1.09 ± 0.13 ^a,b,c^	0.41 ± 0.13	0.83 ± 0.14 ^b^	0.4 ± 0.12	0.59 ± 0.1 ^b^
**Serum BUN (mg/dL)**						
**Sham**	21.8 ± 2.7	22 ± 2.44	21.3 ± 4.17	23.5 ± 3.5	21.5 ± 1.87	22.1 ± 3.37
**IRI**	20.6 ± 1.63	60.5 ± 8.5 ^a^	20.83 ± 3.31	49.3 ± 9.62 ^a^	21.3 ± 2.16	40.1 ± 4.57 ^a^
**ATRA**	22 ± 2.09	42.6 ± 5.7 ^a,b^	23.3 ± 4.8	31.6 ± 2.8 ^b^	23 ± 4.73	27.6 ± 4.41 ^b^
**WJ-MSCs**	21.2 ± 2.56	38.6 ± 4.17 ^a,b^	23 ± 3.57	30 ± 4.1 ^b^	22.17 ± 2.48	26 ± 3.34 ^b^
**ATRA + WJ-MSCs**	22.17 ± 3.31	32.5 ± 4.54 ^a,b,c^	21.17 ± 3.37	27 ± 4.09 ^b^	22.6 ± 3.61	24.3 ± 3.72 ^b^
**Urinary Protein (mg/24 h)**						
**Sham**	1.02 ± 0.14	1.03 ± 0.31	1.03 ± 0.15	1.07 ± 0.09	1.04 ± 0.15	0.99 ± 0.08
**IRI**	1.15 ± 0.37	4.19 ± 0.85 ^a^	1 ± 0.36	3.37 ± 0.66 ^a^	0.95 ± 0.12	2.55 ± 0.95 ^a^
**ATRA**	0.88 ± 0.26	2.95 ± 0.3 ^a,b^	0.96 ± 0.08	1.88 ± 0.33 ^b^	0.8 ± 0.23	1.19 ± 0.25 ^b^
**WJ-MSCs**	0.83 ± 0.21	1.83 ± 0.38 ^b,c^	0.78 ± 0.26	1.21 ± 0.19 ^b^	0.88 ± 0.24	0.84 ± 0.16 ^b^
**ATRA + WJ-MSCs**	0.96 ± 0.16	1.64 ± 0.28 ^b,c^	0.94 ± 0.11	1.05 ± 0.15 ^b^	0.96 ± 0.17	0.87 ± 0.17 ^b^

All data are expressed as mean ± SD. ^a^ Significant vs sham group, ^b^ Significant vs IRI group, ^c^ Significant vs ATRA group and ^d^ Significant vs WJ-MSCs group. One-way analysis of variance (ANOVA) followed by post-hoc multiple comparisons (Tukey test) at *p* ≤ 0.05.

**Table 2:** Effect of pretreatment of WJMSCs with ATRA on markers of oxidative stress (MDA, GSH, SOD and CAT) levels in kidney tissues (n = 6/group).

Group	Day 3	Day 5	Day 7
	**MDA (nmol/g. kidney tissues)**		
**Sham**	15.74 ± 1.5	16.51 ± 1.43	17.16 ± 1.65
**IRI**	68.49 ± 3.68 ^a^	65.75 ± 4.06 ^a^	63.42 ± 2.98 ^a^
**ATRA**	55.44 ± 3.89 ^a,b^	47.67 ± 2.99 ^a,b^	39.03 ± 3.63 ^a,b^
**WJ-MSCs**	51.91 ± 3.22 ^a,b^	43.58 ± 5.00 ^a,b^	32.55 ± 3.59 ^a,b^
**ATRA + WJ-MSCs**	46.32 ± 2.46 ^a,b,c^	38.2 ± 5.94 ^a,b,c^	28.47 ± 2.79 ^a,b,c^
	**GSH (mg/g. kidney tissues)**		
**Sham**	6.5 ± 0.12	6.2 ± 0.26	6.27 ± 0.08
**IRI**	2.1 ± 0.16 ^a^	2.27 ± 0.07 ^a^	2.46 ± 0.06 ^a^
**ATRA**	3.27 ± 0.09 ^a,b^	3.85 ± 0.11 ^a,b^	4.53 ± 0.09 ^a,b^
**WJ-MSCs**	4.13 ± 0.16 ^a,b,c^	5.22 ± 0.07 ^a,b,c^	5.82 ± 0.05 ^a,b,c^
**ATRA + WJ-MSCs**	4.71 ± 0.08 ^a,b,c,d^	5.42 ± 0.08 ^a,b,c^	6.03 ± 0.14 ^a,b,c^
	**SOD (U/g. kidney tissues)**		
**Sham**	204 ± 3.3	201.6 ± 4.28	198.5 ± 4.5
**IRI**	92.9 ± 3.1 ^a^	95.2 ± 4.39 ^a^	98.34 ± 4.53 ^a^
**ATRA**	123.4 ± 4.96 ^a,b^	141.1 ± 3.38 ^a,b^	154.8 ± 5.9 ^a,b^
**WJ-MSCs**	131 ± 5.76 ^a,b^	152.8 ± 3.2 ^a,b,c^	162.7 ± 4.9 ^a,b^
**ATRA + WJ-MSCs**	144.4 ± 5.3 ^a,b,c,d^	158.5 ± 4.5 ^a,b,c^	174.9 ± 7.05 ^a,b,c,d^
	**CAT (U/g. kidney tissues)**		
**Sham**	5.33 ± 0.78	5.1 ± 0.36	4.95 ± 0.16
**IRI**	1.27 ± 0.06 ^a^	1.49 ± 0.1 ^a^	1.68 ± 0.1 ^a^
**ATRA**	1.93 ± 0.18 ^a,b^	2.54 ± 0.09 ^a,b^	3.02 ± 0.1 ^a,b^
**WJ-MSCs**	2.15 ± 0.14 ^a,b^	2.78 ± 0.08 ^a,b^	3.68 ± 0.24 ^a,b,c^
**ATRA + WJ-MSCs**	2.81 ± 0.14 ^a,b,c,d^	3.53 ± 0.13 ^a,b,c,d^	4.15 ± 0.12 ^a,b,c^

All data are expressed as mean ± SD. ^a^ Significant vs sham group, ^b^ Significant vs IRI group, ^c^ Significant vs ATRA group and ^d^ Significant vs WJ-MSCs group. One-way analysis of variance (ANOVA) followed by post-hoc multiple comparisons (Tukey test) at *p* ≤ 0.05.

**Table 3:** Histopathological scores at 3 sacrifice interval time points in each group cortical and medullary histopathological changes in different groups at 3, 5 and 7 days (n = 6/group).

Histological Changes	Day 3		Day 5		Day 7	
	Cortex	Medulla	Cortex	Medulla	Cortex	Medulla
**Tubulo-interstitial damage TID**						
**Sham**	0 (0–0)	0 (0–0)	0 (0–0)	0 (0–0)	0 (0–0)	0 (0–0)
**IRI**	4.5 (3–7) ^a^	4 (3–6) ^a^	4 (3–5) ^a^	3.5 (2–5) ^a^	3 (2–4) ^a^	3 (2–4) ^a^
**ATRA**	3 (2–4) ^a,b^	2.5 (2–4) ^a^	3 (2–4) ^a,b^	3 (2–4) ^a^	2 (0–3) ^a,b^	2 (1–3) ^a,b^
**WJ-MSCs**	2.5 (2–4) ^a,b^	2.5 (2–4) ^a^	3 (2–3) ^a,b^	3 (2–4) ^a^	1.5 (0–2) ^a,b^	1.5 (0–2) ^a,b^
**ATRA + WJ-MSCs**	2.5 (2–4) ^a,b^	2.5 (2–4) ^a,b,c^	1 (1–2) ^a,b,c,d^	1.5 (1–2) ^a,b,c,d^	0 (0–1) ^b,c,d^	1 (0–1) ^b,c,d^
**Regeneration score**						
**Sham**	0 (0–0)	0 (0–0)	0 (0–0)	0 (0–0)	0 (0–0)	0 (0–0)
**IRI**	0 (0–0)	0 (0–0)	0 (0–1)	0 (0–1)	0 (0–1)	0 (0–2)
**ATRA**	0.5 (0–2)	1 (0–2) ^a,b^	2.5 (1–3) ^a,b^	2 (1–3) ^a,b^	3.5 (3–5) ^a,b^	4 (3–5) ^a,b^
**WJ-MSCs**	1.5 (0–3) ^a,b^	1 (0–3) ^a,b^	3 (1–4) ^a,b^	3 (1–4) ^a,b^	3.5 (3–7) ^a,b^	4 (3–6) ^a,b^
**ATRA + WJ-MSCs**	2.5 (1–3) ^a,b^	2.5 (1–3) ^a,b,c^	4 (3–6) ^a,b,c^	4 (3–5) ^a,b,c^	7 (4–8) ^a,b,c,d^	6.5 (4–8) ^a,b,c,d^

All data are expressed as median (minimum–maximum). ^a^ Significant vs sham group, ^b^ Significant vs IRI group, ^c^ Significant vs ATRA group and ^d^ Significant vs WJ-MSCs group. Kruskal-Wallis test followed by Mann–Whitney’s tests at *p* ≤ 0.05.

## Data Availability

All raw data are available on request.
